# The Mosquito Fauna of Arizona: Species Composition and Public Health Implications

**DOI:** 10.3390/insects15060432

**Published:** 2024-06-06

**Authors:** Ndey Bassin Jobe, Nico M. Franz, Murray A. Johnston, Adele B. Malone, Irene Ruberto, John Townsend, James B. Will, Kelsey M. Yule, Krijn P. Paaijmans

**Affiliations:** 1 The Center for Evolution & Medicine, Arizona State University, Tempe, AZ 85281, USA; njobe@asu.edu (N.B.J.); abmalone@asu.edu (A.B.M.); 2 School of Life Sciences, Arizona State University, Tempe, AZ 85281, USA; nico.franz@asu.edu; 3 Department of Entomology, Purdue University, West Lafayette, IN 47907, USA; m.andrew.johnston@gmail.com; 4 Vector Control Division, Maricopa County Environmental Services Department, Phoenix, AZ 85009, USA; john.townsend@maricopa.gov (J.T.); james.will@maricopa.gov (J.B.W.); 5 Arizona Department of Health Services, Phoenix, AZ 85007, USA; irene.ruberto@azdhs.gov; 6 Biodiversity Knowledge Integration Center, Arizona State University, Tempe, AZ 85281, USA; kmyule@asu.edu; 7 Simon A. Levin Mathematical, Computational and Modeling Sciences Center, Arizona State University, Tempe, AZ 85281, USA

**Keywords:** Sonoran desert, medical and veterinary entomology, animal reservoirs, spillover effect, decision making

## Abstract

**Simple Summary:**

Many mosquito species not only serve as a nuisance but also pose a threat to public health by transmitting diseases to both humans and animals. We report an updated list of all known mosquito species identified in Arizona to date. It replaces the most recent lists published about 50 years ago. We also report their collection years, methods, areas/locations, feeding preferences, and the diseases they can or may carry or transmit.

**Abstract:**

Arizona is home to many mosquito species, some of which are known vectors of infectious diseases that harm both humans and animals. Here, we provide an overview of the 56 mosquito species that have been identified in the State to date, but also discuss their known feeding preference and the diseases they can (potentially) transmit to humans and animals. This list is unlikely to be complete for several reasons: (i) Arizona’s mosquitoes are not systematically surveyed in many areas, (ii) surveillance efforts often target specific species of interest, and (iii) doubts have been raised by one or more scientists about the accuracy of some collection records, which has been noted in this article. There needs to be an integrated and multifaceted surveillance approach that involves entomologists and epidemiologists, but also social scientists, wildlife ecologists, ornithologists, representatives from the agricultural department, and irrigation and drainage districts. This will allow public health officials to (i) monitor changes in current mosquito species diversity and abundance, (ii) monitor the introduction of new or invasive species, (iii) identify locations or specific populations that are more at risk for mosquito-borne diseases, and (iv) effectively guide vector control.

## 1. Introduction

Mosquitoes are arthropod vectors that belong to the family Culicidae, comprising 3899 species categorized into 54 genera [1]. Many mosquito species are of medical importance as they can transmit mosquito-borne diseases (MBDs). MBDs pose an enormous threat to global human health worldwide, accounting for about 700 million cases and over 1 million deaths annually [2]. They are caused by parasites (e.g., malaria), viruses (e.g., West Nile and dengue), and worms (lymphatic filariases, heartworm), which are all transmitted by an adult female mosquito to their human or animal host. For example, arboviral diseases such as dengue, Zika, and chikungunya are typically transmitted by *Aedes* species, West Nile virus (WNV) and Saint Louis encephalitis virus (SLEV) by *Culex* species, and the malaria parasite by *Anopheles* species. Some of these diseases (e.g., Zika) can also be transmitted through other routes, such as from female mosquitoes to their offspring (vertical transmission) [3] or via human-to-human interactions through sexual contact [4].

Presently, in Arizona, WNV, SLEV, and dengue are circulating or have been transmitted to humans. In 2021 alone, there were 615 confirmed and 1095 probable human WNV cases, and 127 people who died from the disease in Arizona [5]. The occurrence of SLEV in Arizona is rare, but there was an outbreak in Maricopa County in 2015, which led to 19 confirmed and 3 probable human cases [6]. While autochthonous Zika, chikungunya, and malaria have not been detected in the State recently, locally acquired dengue was reported in Maricopa County in 2022 [7]. Outside Arizona, the local transmission of dengue has been reported in Texas, Hawaii, and Florida [8], Zika in Florida [9] and Texas [10], and more recently (2023), malaria in Florida, Texas, Maryland, and Arkansas [11]. Mosquitoes can also transmit a range of animal diseases, some of which are or have been circulating in Arizona. Examples include WNV in birds (house sparrow, great-tailed grackle, and house finch), which are predominant amplifying hosts for WNV in the State [12,13], Eastern Equine Encephalitis (EEE) in a horse [14], and heartworm in dogs [15].

Some MBDs may currently only circulate in local animal reservoirs [16,17], but could at one point spill-over to humans. A notable example was the human Keystone virus case in Florida in 2016, which is a disease that is normally only found in, e.g., local deer, raccoon, and squirrel populations [16]. Humans (e.g., through tourism, recreational travel, and immigration) can also serve as reservoirs for MBDs when they move between regions when they are infected [18].

While many MBDs have not yet been identified in Arizona, it is important to note that they may circulate but go unnoticed in the State because of (i) an incorrect diagnosis, and (ii) infected individuals not seeking treatment as they are asymptomatic or believe they have a common disease such as the flu [19]. Therefore, it is important to have adequate and comprehensive surveillance systems in place, whereby all key players (animal reservoirs, mosquito vectors, and human hosts) are monitored regularly and where timely information is shared between stakeholders. Here we present a first step in that direction and identify the mosquito species (organized alphabetically by genus) that have been found in Arizona to date, as well as the (potential) risk they pose to both human and animal health. The last comprehensive list of mosquito species in Arizona was published approximately 50 years ago [20]. By presenting this inventory of historic and current records of mosquito species, we aim to enhance the understanding of mosquito biodiversity in Arizona and guide the development of more effective mosquito surveillance and control strategies.

## 2. Materials and Methods

References used in this review were selected from reading peer-reviewed publications identified from searches of PubMed, NCBI, and Google Scholar, from database inception up to 16 February 2024. The search terms used included “mosquito” in conjunction with “Arizona”. Each species identified in Arizona was paired with additional search terms, such as “feeding preference” and “vector competence”, with no language limitations. The taxonomic and nomenclatural conventions for the species listed in this review paper follow [21]. The data records of the species list in this review are from the following sources:

AZDHS—Arizona Department of Health Services. This indicates mosquito collections reported to AZDHS by local vector control agencies across the state.

JB—John Burger. Student in the University of Arizona Department of Entomology in the 1960s. He collected mosquitoes in the State and became a specialist in their taxonomy. He identified many specimens in the University of Arizona Insect Collection (UAIC).

NEON—National Ecological Observatory Network. The NEON Biorepository is operated by Arizona State University in Tempe, AZ [22].

UAIC—University of Arizona Insect Collection. This indicates that at least one mosquito specimen is housed in this collection, and includes records mentioned in [23]. The collection also contains mosquito specimens that JB and others collected.

## 3. Results: Checklist and Review of the Culicidae of Arizona (Insecta: Diptera)

### 3.1. Genus Aedes Meigen 1818

There have been 23 species of *Aedes* identified in Arizona (Table 1). *Aedes aegypti* (Linnaeus) and *Aedes albopictus* (Skuse) can vector a range of arboviruses to humans, including Zika, dengue, and chikungunya (Table 1).

In Arizona, the most common *Aedes* species of medical importance is *Aedes aegypti* (Table 1). Over the years, *Aedes aegypti* has also been the most abundant *Aedes* species reported to AZDHS by local vector control agencies. The collection of *Aedes albopictus* (also known as the Asian tiger mosquito) in 2008 in Maricopa County is noteworthy since this is the most invasive mosquito species in the world [24]. This collection was because of a complaint about mosquitoes in an office in Chandler, which led to an investigation. It was discovered that an employee brought back a ‘volcano plant’ from Hawaii to the office, and *Aedes albopictus* was being produced from eggs laid on the volcanic rock attached to the plant. The Maricopa County Environmental Services Department Vector Control Division confiscated the plant, contained and isolated the eggs, and ultimately reared about 60 *Aedes albopictus* (personal communication, James B. Will and John Townsend, Maricopa County Environmental Services Department). *Aedes albopictus* is a competent vector for various arboviruses, including Chikungunya virus [25], dengue virus [26], and Zika virus [27]. While this invasive species has not recently been found in Arizona, it has established itself in several counties in the neighboring State of California [28].

Interestingly, while *Culex* Linnaeus species are typically held responsible for the transmission of WNV (see below), *Aedes albopictus* can be a competent vector of the disease under laboratory conditions [29]. WNV has also been isolated from field-collected (i) *Aedes albopictus* in Baltimore, MD, in 2015 and 2017 [30], (ii) *Aedes aegypti* and *Aedes epactius* (Dyar & Knab) in Chihuahua (shares border with Texas, USA), Mexico, in 2021 [31], (iii) *Aedes taeniorhynchus* (Wiedemann) in eastern Puerto Rico, in 2007 [32], and (iv) *Aedes vexans* (Meigen) in New Jersey, in 2001 [33].

It has been shown that *Aedes sollicitans* (Walker) can transmit EEE to animals (e.g., chickens and horses) under laboratory conditions [34,35,36]. *Aedes sollicitans* and *Aedes vexans* have also been implicated as vectors of EEE to humans during an outbreak in New Jersey [37].

*Aedes vexans* can transmit heartworm disease to dogs under laboratory conditions [38]. Dog heartworm disease has also been isolated from field-collected *Aedes sollicitans* and *Aedes taeniorhynchus*, in the Yucatan, southeastern Mexico, in 2007 [39].

Some other *Aedes* species that have been collected in the State are not (yet) associated with disease transmission and have also not been collected recently (but note that this may be due to the collection methods and protocols in place, see Discussion). These species include *Aedes burgeri* (Zavortink), *Aedes monticola* (Belkin & McDonald), *Aedes muelleri* (Dyar), *Aedes papago* (Zavortink), *Aedes pullatus* (Coquillett), and *Aedes purpureipes* (Aitken).

**Table 1:** List of *Aedes* species.

Collection Year	Collection Method	Collection Area (by County)	Feeding Preference	Diseases They Can/May Transmit/Carry
***Aedes aegypti* (Linnaeus 1762)**				
1931–1943, 1994–2022 (UAIC)	Unknown	Apache Pima	Humans, dogs, swine [40] Birds [41]	Yellow fever [42,43] ^a, †^ Zika virus [44] ^a, †^ [45,46] ^b,^^†††^ dengue virus [47] ^a, †††^ [48] ^a, †^ [49,50] ^b, †††^ Chikungunya virus [51] ^a, †, †††^ [52] ^b, †††^ West Nile virus [31] ^b, †††^
On or before 1946 [53]	Unknown	Unknown		
On or before 1997 [54]	CO_2_-baited traps and hay-infused enhanced oviposition traps [54]	Unknown		
2003–2023 (AZDHS)	A variety of CO_2_ traps and/or BG-Sentinel and/or Oviposition traps	Apache Cochise Coconino Gila Graham Greenlee Maricopa Mohave Pima Pinal Santa Cruz Yavapai Yuma		
2018, 2021, 2022 (NEON)	CDC CO_2_ light traps baited with dry ice	Pima		
2021 [55]	BG-pro trap baited with dry ice	Maricopa (Tempe)		
***Aedes albopictus* (Skuse 1895)**				
2008–2009 (Maricopa County Vector Control)	Collected from a volcano plant brought to Chandler, Arizona, from Hawaii.	Maricopa	Humans [56]	West Nile virus [29] ^a, †^ [30] ^b^ Chikungunya virus [25] ^a, †, †††^ [57] ^b, †††^ dengue virus [26] ^a, †^ [58] ^b, †††^ Zika virus [27] ^a, †, †††^ [46] ^b, †††^
***Aedes burgeri* Zavortink 1972**				
1964 (UAIC)	Unknown (labelled as *Ae. kompi*)	Pima	Unknown	Unknown
1964 [59,60]	JB collected pupae (as *Ae. kompi*) from a tree hole [59,60]	Santa Cruz		
***Aedes cataphylla* Dyar 1916**				
On or before 1956 [61]	Unknown	Coconino	Humans [23]	James Town Canyon virus [62] ^b^
1964 (UAIC)	Unknown	Apache		
On or before 1973 [23]	Unknown	Coconino Apache		
***Aedes dorsalis* (Meigen 1830)**				
1944 (UAIC)	Unknown	Yuma	Humans [23,63]	California encephalitis virus [63] ^b^ Western equine encephalitis virus [63] ^b^ [64] ^b, †††^
On or before 1956 [61]	Unknown	Apache Navajo Pima Yuma Yavapai		
On or before 1973 [23]	Unknown	Apache Navajo Pima Santa Cruz Yuma		
2012–2017, 2019 (AZDHS)	A variety of CO_2_ traps and/or BG-Sentinel and/or Oviposition traps	Yuma Yavapai La Paz Navajo Cochise Pima		
***Aedes epactius* Dyar & Knab 1908**				
1963–1964 (UAIC)	Unknown	Cochise Pima	Humans [61]	Jamestown Canyon virus [65] ^a, ††^ West Nile virus [31] ^b, †††^
2014 (AZDHS)	A variety of CO_2_ traps and/or BG-Sentinel and/or Oviposition traps	Yuma		
2016, 2021 (NEON)	CDC CO_2_ light traps baited with dry ice	Pima		
***Aedes fitchii* (Felt & Young 1904)**				
On or before 1956 [61]	Unknown	Coconino	Humans [66]	Aleutian disease virus [67] ^a^ Snowshoe hare virus [68] ^b^
On or before 1973 [23]	Unknown	Coconino		
***Aedes hexodontus* Dyar 1916** **^♦^**				
2007 (UAIC)	Unknown	Coconino	Unknown	Jamestown Canyon virus [62,69] ^b^
2013 (AZDHS)	A variety of CO_2_ traps and/or BG-Sentinel and/or Oviposition traps	Coconino		
***Aedes implicatus* Vockeroth 1954**				
1967 [70]	Larval collection	Greenlee	Unknown	Snowshoe hare virus [71] ^c^
***Aedes increpitus* Dyar 1916**				
On or before 1974 [20]	Unknown	Unknown	Livestock (e.g., cattle), wildlife (e.g., deer) [72] Mammals [66]	California encephalitis virus [73] ^a^
2019 (AZDHS)	A variety of CO_2_ traps and/or BG-Sentinel and/or Oviposition traps	Coconino		
***Aedes monticola* Belkin & McDonald 1957** *				
On or before 1957 [23]	Unknown	Cochise Graham Pima Santa Cruz	Humans [23]	Unknown
2018, 2019, 2022 (NEON)	CDC CO_2_ light traps baited with dry ice	Pima		
Unknown (UAIC)	Unknown	Unknown		
***Aedes muelleri* Dyar** **1920**				
1917 [63]	Caught while biting a human	Head of Indian Creek in the Chiricahua Mountains in Arizona at 6100 feet elevation	Unknown	Unknown
1922, 1928 [61]	Unknown	Cochise Santa Cruz		
1964–1968 (UAIC)	Unknown	Cochise Pima		
On or before 1973 [23]	Unknown	Cochise Pima Santa Cruz		
***Aedes nigromaculis* (Ludlow 1906)**				
On or before 1956 [61]	Unknown	Navajo Yavapai	Humans and animals [23]	Western equine encephalitis [74] ^b^
1964, 2010–2015 (UAIC)	Unknown	Pima		
2012–2015, 2017 (AZDHS)	A variety of CO_2_ traps and/or BG-Sentinel and/or Oviposition traps	Pima Coconino Navajo		
2018 (NEON)	CDC CO_2_ light traps baited with dry ice	Pima		
***Aedes papago* Zavortink 1970**				
On or before 1973 [23]	Unknown	Pima	Unknown	Unknown
2016–2018, 2021–2022 (NEON)	CDC CO_2_ light traps baited with dry ice	Pima		
Unknown (UAIC)	Unknown	Pima		
***Aedes pullatus* (Coquillett** **1904)**				
1967 [70]	Larval collection	Greenlee	Unknown	Unknown
On or before 1974 [20]	Unknown	Unknown		
***Aedes purpureipes* Aitken** **1941**				
On or before 1956 [61]	Unknown	Pima Santa Cruz	Humans [23]	Unknown
1996–2022 (UAIC)	Unknown	Cochise Pima Pinal Yavapai		
2012–2013, 2015–2017, 2019 (AZDHS)	A variety of CO_2_ traps and/or BG-Sentinel and/or Oviposition traps	Pima Cochise Maricopa Pinal Santa Cruz Gila		
2016–2022 (NEON)	CDC CO_2_ light traps baited with dry ice	Pima		
***Aedes sollicitans* (Walker 1856)**				
On or before 1956 [61]	Unknown	Yuma	Humans [23] Birds and mammals [75]	West Nile virus [76] ^b, †††^ Port Bolivar virus [77] ^b, †††^ Cache Valley virus [78] ^a, ††^ [79] ^b^ Eastern equine encephalitis virus [35] ^a, b, ††^ Dog heartworm disease [39] ^b, †††^
***Aedes taeniorhynchus* (Wiedemann 1821)**				
1962 [80]	A single female, taken in a biting collection at Yuma Test Station	Yuma	Humans and Birds [81] Mammals, reptiles and birds [82]	West Nile virus [83] ^a, ††^ [32] ^b, †††^ Venezuelan equine encephalitis virus [84] ^a, ††, †††^ Dog heartworm disease [39] ^b, †††^
***Aedes thelcter* Dyar** **1918**				
On or before 1990 [85]	Unknown	Yuma	Unknown	Unknown
2000–2022 (UAIC)	Unknown	Pima		
2012–2017, 2019 (AZDHS)	A variety of CO_2_ traps and/or BG-Sentinel and/or Oviposition traps	Pima		
2016, 2018, 2021 (NEON)	CDC CO_2_ light traps baited with dry ice	Pima		
***Aedes trivittatus* (Coquillett 1902)**				
1953–1964 (UAIC)	Unknown	Cochise Navajo Santa Cruz	Mammals (humans, deer, cat, horse, cow) and avian [86]	Trivittatus virus [87] ^a, ††^ [88] ^c^ West Nile virus [89] ^a^ Shunk river virus [90] ^b, †††^
On or before 1956 [61]	Unknown	Apache Greenlee Cochise Santa Cruz Gila		
2014–2017, 2019 (AZDHS)	A variety of CO_2_ traps and/or BG-Sentinel and/or Oviposition traps	Apache Cochise Greenlee Gila Navajo Santa Cruz Coconino		
2016, 2017, 2018, 2022 (NEON)	CDC CO_2_ light traps baited with dry ice	Pima		
***Aedes varipalpus* (Coquillett 1902)**				
On or before 1956 [61]	Unknown	Cochise Coconino Graham Pima	Humans [66]	California Encephalitis virus [91]
1963 (UAIC)	Unknown	Gila		
2015 (AZDHS)	Unknown	Navajo		
***Aedes ventrovittis* Dyar** 1916 **				
1964 (UAIC)	Unknown	Greenlee	Unknown	Unknown
On or before 1973 [23]	Unknown	Greenlee		
***Aedes vexans* (Meigen 1830)**				
1938–2022 (UAIC)	Unknown	Cochise Navajo Pima Yuma	Humans [23,66] Other mammals (deer, horses, cats) and birds (American robin) [92]	West Nile virus [93] ^a, †^ [33] ^b^ St. Louis encephalitis virus [94] ^a^ Zika virus [95] ^a, †, †††^ [96] ^b, †††^ Eastern equine encephalomyelitis virus [97] ^b, †††^ Dog heartworm disease [38] ^a, ††^ [98] ^b, †††^
On or before 1956 [61]	Unknown	Apache Cochise Graham Greenlee Maricopa Pima Pinal Yavapai Yuma		
2012–2017, 2019–2020 (AZDHS)	A variety of CO_2_ traps and/or BG-Sentinel and/or Oviposition traps	Cochise Coconino Gila Graham La Paz Maricopa Mohave Navajo Pima Pinal Santa Cruz Yavapai Yuma		
2017, 2022 (NEON)	CDC CO_2_ light traps baited with dry ice	Pima		

♦ One or more scientists in the State have expressed doubts about the accuracy of this collection record. * *Ae. monticola* (Belkin & McDonald) is cataloged as *Ae. varipalpus* in the UAIC collection because of the difficulty in morphologically distinguishing it from *Ae. varipalpus*. ** *Ae. ventrovittis* was on the 1973 list [23] but removed from the 1974 list without explanation [20]. However, one female that was collected and identified as *Ae. ventrovittis* by JB remains in the UAIC collection. ^a^ Vector competence study under laboratory conditions. ^b^ Virus isolated from field-collected specimens. ^c^ Transovarial transmission. ^†^ Detection of virus in the saliva of mosquito post-infection. ^††^ Evidence of transmission to animals by an infected mosquito. ^†††^ Disseminated infection (i.e., detection of virus in body, legs, and/or wings of mosquito).

### 3.2. Genus Anopheles Meigen 1818

There are five species of *Anopheles* that have been identified in Arizona, which are *Anopheles franciscanus* (McCracken), *Anopheles freeborni* (Aitken), *Anopheles hermsi* (Barr & Guptavanij), *Anopheles judithae* (Zavortink), and *Anopheles pseudopunctipennis* (Theobald). Mosquitoes of the *Anopheles* genus can transmit malaria parasites to humans [99]. They can also transmit filarial parasites, causing human lymphatic filariasis [100] and arboviruses, such as Venezuelan equine encephalitis virus [101].

Three of the anopheline species that have been found in the State (*Anopheles freeborni*, *Anopheles hermsi*, and *Anopheles pseudopunctipennis*) can transmit or carry human malaria (Table 2). While human malaria is currently not locally transmitted in Arizona, historical records show it was a public health concern in the 19th century [102]. Public health scientists have suggested that *Anopheles hermsi* may have played an important role in its transmission during that period. Laboratory tests have shown that *Anopheles hermsi* is susceptible to *Plasmodium vivax* [103], suggesting that this species may have been historically implicated in malaria outbreaks that occurred in California and New Mexico [102]. This raises concerns for the potential introduction of *P. vivax* by travelers from regions where the parasite is endemic, such as India [104], to Arizona, where *An. hermsi* is present (Table 2). *Anopheles hermsi* is not a known vector of *Plasmodium falciparum* (the most deadly and prevalent malaria parasite [105]); however, further testing is needed to evaluate its potential role in the transmission of this malaria parasite. The last documented collection record of *Anopheles hermsi* reported to AZDHS (Table 2) was in 2014. Again, it is important to note that this may be due to the current trapping methods and surveillance strategies (see Discussion).

Finally, *Anopheles pseudopunctipennis* may be an important species to monitor. It was recently collected in Pima County in 2022 (Table 2), is anthropophilic, and has been implicated in *P. vivax* malaria [106] and Venezuelan equine encephalitis virus transmission [107].

**Table 2:** List of *Anopheles* species.

Collection Year	Collection Method	Collection Area (by County)	Feeding Preference	Diseases They Can/May Transmit
***Anopheles franciscanus* McCracken 1904**				
1934–2022 (UAIC)	Unknown	Cochise Graham Maricopa Pima Santa Cruz	Large mammals (Horse, cow sheep) and small animals (duck, turkey, rabbit, guinea pig) [108]	Unknown
On or before 1956 [61]	Unknown	Cochise Coconino Gila Graham Greenlee Maricopa Mohave Navajo Pima Pinal Santa Cruz Yavapai Yuma		
2012–2017, 2019 (AZDHS)	A variety of CO_2_ traps and/or BG-Sentinel and/or Oviposition traps	Cochise Coconino Gila Graham Greenlee Maricopa Mohave Navajo Pima Pinal Santa Cruz Yavapai Yuma		
2016, 2017, 2020–2022 (NEON)	CDC CO_2_ light traps baited with dry ice	Pima		
***Anopheles freeborni* Aitken 1939** **^♦^**				
2013, 2017 (AZDHS)	A variety of CO_2_ traps and/or BG-Sentinel and/or Oviposition traps	Santa Cruz Pinal	Large mammals [66]	Malaria [109] ^a, †^ Northway serotype virus [110] ^b^
***Anopheles hermsi* Barr and Guptavanij 1989**				
1995 and 1997 [102]	Unknown	Cochise Navajo Santa Cruz	Humans [102]	Malaria [103] ^a^
2004–2010, 2012, 2014 (AZDHS)	A variety of CO_2_ traps and/or BG-Sentinel and/or Oviposition traps	Yuma Cochise Yavapai		
Unknown (UAIC)	Unknown	Cochise Pima		
***Anopheles judithae* Zavortink 1969**				
1964 (UAIC)	Unknown	Cochise	Unknown	Unknown
On or before 1969 [111]	Unknown	Unknown		
On or before 1973 [23]	Unknown	Cochise Maricopa Pima Santa Cruz Yavapai		
***Anopheles pseudopunctipennis* Theobald 1901**				
2015, 2017 (AZDHS)	A variety of CO_2_ traps and/or BG-Sentinel and/or Oviposition traps	Pima	Humans [112]	Venezuelan equine encephalitis virus [107] ^b^ Malaria [113] ^b^
2016, 2018–2022 (NEON)	CDC CO_2_ light traps baited with dry ice	Pima		

**^♦^** One or more scientists in the State have expressed doubts about the accuracy of this collection record. ^a^ Vector competence study under laboratory conditions. ^b^ Virus or parasite isolated from field-collected specimens. ^†^ Detection of the parasite in the saliva of mosquito post-infection.

### 3.3. Genus Culex Linnaeus 1758

There are 14 species of *Culex* identified in Arizona. Mosquitoes of this genus can carry or transmit arboviruses, such as WNV [76,114], SLEV [94], and Western equine encephalitis (WEE) [23,115], but also Zika virus [96], which is commonly associated with *Aedes* species (see below).

The most common *Culex* species of medical importance (vectors of, e.g., WNV & SLEV) are *Culex quinquefasciatus* (Say) and *Culex tarsalis* (Coquillett) (Table 3). These have also been the most abundant species reported to AZDHS in recent years. Many other *Culex* species identified in the State can carry and/or transmit WNV (Table 3). WNV is endemic to Arizona, with sporadic outbreaks of the disease [116,117], including the largest documented outbreak of the disease in a single county in the history of the United States in 2021 [118].

While mosquitoes of the genus *Aedes*, particularly *Aedes aegypti* and *Aedes albopictus*, are commonly implicated with the transmission of Zika [119,120], the arbovirus has been found in field-collected *Culex coronator* (Dyar & Knab), *Culex quinquefasciatus*, and *Culex tarsalis* [96].

Finally, *Culex erraticus* (Dyar & Knab) has been incriminated as a laboratory vector of reptilian malaria caused by *Plasmodium floridense* [121].

**Table 3:** List of *Culex* species.

Collection Year	Collection Method	Collection Area (by County)	Feeding Preference	Diseases They Can/May Transmit
***Culex abominator* Dyar and Knab 1909** **^♦^**				
2012–2014 (AZDHS)	A variety of CO_2_ traps and/or BG-Sentinel and/or Oviposition traps	Pima Yavapai Yuma Pinal	Unknown	Unknown
***Culex apicalis* Adams 1903**				
1930–1963 (UAIC)	Unknown	Cochise Pima	Reptiles, amphibians, and birds [66]	Unknown
On or before 1956 [61]	Unknown	Apache Coconino Maricopa Navajo Pima Yavapai		
***Culex arizonensis* Bohart 1949**				
On or before 1956 [61]	Unknown	Pima Santa Cruz Yavapai	Unknown	Unknown
1961–1964 (UAIC)	Unknown	Pima Santa Cruz		
***Culex coronator* Dyar and Knab** **1906**				
1930–1963, 1996–2021 (UAIC)	Unknown	Unknown	Mammals and avian [122]	West Nile virus [114] ^a, †, †††^ [76] ^b, †††^ Zika virus [96] ^b, †††^ St. Louis Encephalitis virus [94] ^a^
On or before 1956 [61]	Unknown	Cochise Pima		
2012–2015, 2019 (AZDHS)	A variety of CO_2_ traps and/or BG-Sentinel and/or Oviposition traps	Pima		
***Culex erraticus* (Dyar and Knab 1906)**				
2012–2021 (AZDHS)	A variety of CO_2_ traps and/or BG-Sentinel and/or Oviposition traps	Yuma	Humans and other mammals, birds, amphibians [123]	West Nile virus [76] ^b, †††^ Eastern equine encephalomyelitis virus [97] ^b, †††^ Eastern equine encephalitis [124] ^b^ Reptilian malaria [121] ^a, †^
Unknown (UAIC)	Unknown	Yuma		
*Culex erythrothorax* Dyar 1907 *				
On or before 1956 [61]	Unknown	Cochise	Humans and birds [23]	West Nile virus [125] ^b, †††^ [93] ^a, †^
On or before 1973 [23]	Unknown	Cochise Santa Cruz Yuma		
1998–2023 (UAIC)	Unknown	Pima		
2012–2021 (AZDHS)	A variety of CO_2_ traps and/or BG-Sentinel and/or Oviposition traps	Apache Cochise Coconino Graham Imperial Maricopa Mohave Navajo Pima Pinal Santa Cruz Yavapai Yuma		
***Culex nigripalpus* Theobald 1901**				
1962 (UAIC)	Unknown	Santa Cruz	Mammals and avian [122]	West Nile virus [76] ^b, †††^ St. Louis encephalitis virus [126] ^b, †††^ Eastern equine encephalitis virus [127] ^b^
***Culex pipiens* Linnaeus 1758** **^♦^**				
2012–2013, 2015, 2021 (AZDHS)	A variety of CO_2_ traps and/or BG-Sentinel and/or Oviposition traps	Yuma	Birds [128] Humans [129]	West Nile virus [130] ^b, †††^ Usutu virus [131] ^a, †^ Avian malaria [132] ^b, †††^
***Culex quinquefasciatus* Say 1823**				
1920–2023 (UAIC)	Unknown	Graham Pima	Mammals and avian [122]	West Nile virus [133] ^a, †, †††^ [31,76] ^b, †††^ Zika virus [96] ^b, †††^ St. Louis encephalitis virus [134] ^a^
On or before 1956 [61]	Unknown	Cochise Graham Maricopa Pima Pinal Santa Cruz Yuma		
2010, 2012–2021 (AZDHS)	A variety of CO_2_ traps and/or BG-Sentinel and/or Oviposition traps	Pinal Pima Maricopa Mohave Yavapai Yuma Cochise La Paz Santa Cruz Coconino Graham Navajo Gila Apache		
2016, 2019, 2020, 2021, 2022 (NEON)	CDC CO_2_ light traps baited with dry ice	Pima		
In 2021 [55]	BG-pro trap baited with dry ice	Maricopa (Tempe)		
***Culex restuans* Theobald 1901**				
On or before 1956 [61]	Unknown	Santa Cruz	Birds, humans and other vertebrates [135]	West Nile Virus [136] ^a, †, †††^ La Crosse virus [137] ^a, †^
2015 (AZDHS)	A variety of CO_2_ traps and/or BG-Sentinel and/or Oviposition traps	Pima		
*Culex stigmatosoma* Dyar 1907 **				
On or before 1956 [61]	Unknown	Cochise	Birds, mammals and reptiles [138]	West Nile virus [93] ^a, †^
On or before 1973 [23]	Unknown	Cochise Pima Pinal Santa Cruz Yuma		
***Culex tarsalis* Coquillett** **1896**				
1935–2023 (UAIC)	Unknown	Pima	Birds, cattle, horses, and humans [135]	West Nile virus [93] ^a, †^ [31] ^b, †††^ Zika virus [96] ^b, †††^ St. Louis Encephalitis virus [94] ^a^ [134] ^a^ Western equine encephalomyelitis [139] ^a^ Western equine encephalitis [115] ^a^ [64] ^b^
On or before 1956 [61]	Unknown	Collected in large numbers in every county at the time except Greenlee		
2012–2021 (AZDHS)	A variety of CO_2_ traps and/or BG-Sentinel and/or Oviposition traps	Apache Cochise Greenlee La Paz Maricopa Mohave Pima Pinal Yavapai Yuma Coconino Gila Graham Navajo Santa Cruz Imperial		
2016–2022 (NEON)	CDC CO_2_ light traps baited with dry ice	Pima		
In 2021 [55]	BG-pro trap baited with dry ice	Maricopa (Tempe)		
***Culex territans* Walker 1856**				
On or before 1956 [61]	Unknown	Cochise	Amphibians, reptiles, humans and other mammals [66]	Unknown
2015, 2017 (AZDHS)	A variety of CO_2_ traps and/or BG-Sentinel and/or Oviposition traps	Pima		
***Culex thriambus* Dyar 1921**				
1953–2022 (UAIC)	Unknown	Pima Maricopa Cochise Santa Cruz Yuma Mohave	Birds [140]	West Nile virus [140] ^b^
On or before 1956 [61]	Unknown	Apache Cochise Coconino Gila Greenlee Maricopa Mohave Navajo Pima Pinal Santa Cruz Yavapai		
2012–2013, 2015–2017, 2019 (AZDHS)	A variety of CO_2_ traps and/or BG-Sentinel and/or Oviposition traps	Pima Coconino		

^♦^ One or more scientists in the State have expressed doubts about the accuracy of this collection record. * Culex erythrothorax (Dyar) was listed as *Culex pipiens quinquefasciatus* (Say) in the 1973 and 1974 lists of mosquitoes of Arizona [20,23]. ** Culex stigmatosoma (Dyar) was listed as *Culex peus* (Speiser), a now suppressed name, in the 1973 and 1974 lists of mosquitoes of Arizona [20,23]. ^a^ Vector competence study under laboratory conditions. ^b^ Virus isolated from field-collected specimens. ^†^ Detection of virus in the saliva of mosquito post-infection. ^†††^ Disseminated infection (i.e., detection of virus in body, legs, and/or wings of mosquito).

### 3.4. Genus Culiseta Felt 1904

There are three species of *Culiseta* identified in Arizona as outlined in Table 4. Mosquitoes of this genus can transmit arboviruses, such as WNV, WEE, and SLEV [93,94,115,141]. All three species found in Arizona feed on mammals, including humans, and could serve as a potential vector for the transmission of arboviral diseases that are already present in the State, such as WNV and SLEV.

While mosquitoes of the *Culex* genus are more commonly associated with and screened for diseases like WNV and SLEV, *Culiseta incidens* (Thomson) and *Culiseta inornata* (Williston) have been successfully infected with WNV and SLEV in the laboratory (Table 4). While these species have been collected recently in the State, they are not typically screened for the presence of arboviruses (e.g., WNV) by public health departments.

Finally, *Culiseta incidens* and *Culiseta inornata* are confirmed laboratory vectors of WEE [115]. The virus has also been isolated from field-collected *Culiseta inornata* in southern Saskatchewan, Canada, in 1962 [64].

**Table 4:** List of *Culiseta* species.

Collection Year	Collection Method	Collection Area (by County)	Feeding Preference	Diseases They Can/May Transmit
***Culiseta incidens* (Thomson 1869)**				
1917–2022 (UAIC)	Unknown	Cochise Maricopa Navajo Pima Pinal	Mammals, including humans [23,66]	West Nile virus [141] ^a^ St. Louis encephalitis virus [94] ^a^ Western equine encephalitis [115] ^a, ††^
On or before 1956 [61]	Unknown	Graham Greenlee Pinal		
On or before 1973 [23]	Unknown	All counties at the time except Pinal and Graham		
2013, 2016–2017, 2019 (AZDHS)	A variety of CO_2_ traps and/or BG-Sentinel and/or Oviposition traps	Coconino Yavapai Coconino Pima		
2020 (NEON)	CDC CO_2_ light traps baited with dry ice	Pima		
***Culiseta inornata* (Williston 1893)**				
1920–1989 (UAIC)	Unknown	Cochise Maricopa Mohave Pima Santa Cruz Yuma	Humans and other mammals (cattle) [66]	West Nile virus [93] ^a, †^ St. Louis encephalitis virus [94] ^a^ Western equine encephalitis [115] ^a^ [64] ^b, †††^
On or before 1956 [61]	Unknown	Gila Graham Greenlee		
On or before 1973 [23]	Unknown	All counties at the time except Gila, Greenlee and Graham		
2012–2017, 2019 (AZDHS)	A variety of CO_2_ traps and/or BG-Sentinel and/or Oviposition traps	Coconino Pima Yavapai Yuma Cochise Gila Maricopa Mohave Navajo Apache		
2018–2019, 2021–2022 (NEON)	CDC CO_2_ light traps baited with dry ice	Pima		
In 2021 [55]	BG-pro trap baited with dry ice	Maricopa (Tempe)		
*Culiseta particeps* (Adams 1903) *				
On or before 1956 [61]	Unknown	Santa Cruz	Humans and other large mammals [23,66]	Unknown
1963–1997 (UAIC)	Unknown	Cochise Pima		
On or before 1973 [23]	Unknown	Cochise Pima Santa Cruz		

* Culiseta particeps (Adams) was listed as *Culiseta maccrackenae* (Dyar & Knab) elsewhere [61]. Although this species feeds on both humans and other animals, it has not been associated with any diseases. ^a^ Vector competence study under laboratory conditions. ^b^ Virus isolated from field-collected specimens. ^†^ Detection of virus in the saliva of mosquito post-infection. ^††^ Evidence of transmission to animals by an infected mosquito. ^†††^ Disseminated infection (i.e., detection of virus in body, legs, and/or wings of mosquito).

### 3.5. Genus Orthopodomyia Theobald 1904

There are two species of *Orthopodomyia* identified in Arizona (Table 5). One of these species (*Orthopodomyia signifera* (Coquillett)) has been implicated in the transmission of EEE and WEE [142]. Although these two arboviruses are not currently circulating in the State, a horse recently tested positive for EEE [14], which suggests that monitoring this genus, in addition to, e.g., *Aedes sollicitans*, *Aedes vexans*, and *Culex tarsalis*, may be warranted. There are also no recent records of this genus in Arizona, which again may be attributed to surveillance systems currently in place (see Discussion).

### 3.6. Genus Psorophora Robineau-Desvoidy 1827

There are six species of *Psorophora* identified in Arizona (Table 6). *Psorophora confinnis* (Lynch-Arribalzaga) has been implicated as a probable vector of VEEV during epidemics and epizootics observed in Mexico, Venezuela, the southern United States, and Guatemala [143]. Additionally, WNV has also been detected in field-collected *Psorophora columbiae* (Dyar & Knab) and *Psorophora signipennis* (Coquillett) [116,144]. As *Culex* species are more commonly associated with and screened for WNV, *Psorophora* species are not included in the routine WNV surveillance by the public health departments in the State (see Discussion). Finally, field-collected *Psorophora columbiae* (Dyar & Knab) has been found infected with dog heartworm in 2010, in Payne County, Oklahoma [145].

### 3.7. Genera Toxorhynchites Theobald 1901 and Uranotaenia Lynch Arribálzaga 1891

*Toxorhynchites moctezuma* (Dyar & Knab), *Uranotaenia anhydor* (Dyar), and *Uranotaenia sapphirina* (Osten Sacken) have been identified in Arizona (Table 7). These species have no known association with disease transmission in humans and/or animals.

*Toxorhynchites moctezuma* are non-blood-feeding mosquitoes known for their larval predatory behavior (their larvae feed on the larvae of other mosquito species) [150]. Therefore, they may be used as a biological control for other mosquito species that are responsible for the transmission of MBDs.

There are no documented collection records of *Uranotaenia anhydor* since 1956 [61], which may again be attributed to surveillance systems currently in place (see Discussion). However, the collection of *Uranotaenia sapphirina* (Osten Sacken) was last reported to AZDHS in 2015 (Table 7).

## 4. Discussion

There is a total of 56 mosquito species that have been identified in Arizona to date, many of which can transmit disease to humans and/or other animals. This list serves as a foundational resource for understanding the mosquito diversity in the State. It is meant to guide both surveillance (e.g., target species in surveillance programs and molecular detection of diseases in different mosquito species) and research (e.g., improving our understanding of mosquito feeding preference and vector competence) agendas. While it is a good starting point for continued discussions about mosquito surveillance practices in the State, there are a few caveats, which we list below.

First, it is important to note that the morphological identification of some species may have been inaccurate, since some species are morphologically indistinguishable. To illustrate, the 1973 list included *Anopheles barberi* (Coquillett) [23], which may actually have been *Anopheles judithae*. *Anopheles hermsi* was cataloged as *Anopheles freeborni* in the 1973 and 1974 lists [20,23], which may have been due to their similar morphologies [102]. While *Aedes infirmatus* was initially included on the 1973 and 1974 lists of mosquitoes found in Arizona [20,23], it was noted as a misidentified *Aedes trivittatus* a few years later [151]. Currently, many health departments use dichotomous mosquito identification keys, which may result in misidentification. This can be overcome by species-specific molecular identification tools, such as internal transcribed spacer 2 (ITS2) of ribosomal DNA (rDNA) [152] and mitochondrial cytochrome C oxidase subunit 1 (CO1) gene-based DNA barcoding [153]. This will target specific rDNA or DNA sequences that are unique to individual mosquito species and allow for highly specific identification, even among closely related species with morphological similarities. Also, it is worthwhile to note that dichotomous keys are sometimes the most cost-effective mosquito identification method for some health departments and can be an effective identification tool if personnel are properly trained. Accurately identifying species is important in assessing MBD transmission risks and developing appropriate vector control strategies, since different species may differ in their competency in transmitting diseases.

Second, there is often no information on the collection/sampling methods for many of the mosquito species presented here. When information is available, CO_2_-baited traps (often using dry ice) are most commonly used in the State. Maricopa County, the largest county in Arizona that includes the city of Phoenix, uses EVS (Encephalitis-Virus-Surveillance) traps more than other traps [154]. Although these traps collect *Aedes aegypti* [155,156,157], there are other trap types that may be more efficient in trapping *Aedes* species, such as the BG-Sentinel trap [157]. The same applies to anopheline species, where, e.g., CDC light traps [158] and Mosquito Magnet Patriot Mosquito traps [159] may be more effective in capturing this genus. In addition, other entomological indicators that can be used to guide vector control, such as (i) larval habitat preference (assessed through, e.g., larval dipping [160] or the use of environmental DNA [161]), (ii) indoor resting densities (assessed through, e.g., indoor resting collections and window exit traps), and (iii) the time of mosquito activity (assessed through, e.g., hourly switching of collection nets or rotator traps) are better assessed using different sampling methods [160]. Using the appropriate mosquito sampling methods in surveillance programs will ensure that the obtained data are reliable and relevant for public health decision-making, by identifying the appropriate control tools to optimally target vectors of medical importance [162].

Local Vector Control agencies should adopt a multifaceted mosquito surveillance approach that encompasses not only monitoring mosquito abundance in various counties but also investigating other ecological and behavioral relevant factors, such as breeding site selection, host preferences, flight ranges, and insecticide resistance status. For example, knowledge of their preferred breeding sites and areas allows for more effective and targeted larviciding efforts. And—as outlined in Table 1, Table 2, Table 3, Table 4, Table 5, Table 6 and Table 7—there is a large variation in the feeding preference (i.e., host preference) between the different mosquito species, which determines the disease transmission risk to both humans and animals (i.e., an anthropophilic vector poses a larger threat to human health than a zoophilic vector and vice versa for animal health). It is also important to understand the flight ranges of the different species to create and implement spatially informed vector control strategies [163]. Resistance to many classes of insecticides has been observed in a range of mosquito species (e.g., *Aedes*, *Culex*, *Anopheles*), which affects vector control efforts [164,165,166,167] and has been reported for the main vector species (*Culex quinquefasciatus*, *Culex tarsalis*, and *Aedes aegypti*) by AZDHS and partners. Continuously monitoring insecticide resistance and developing an insecticide resistance management plan that also includes some of the other vector species listed in this paper allows us to ensure that current and future insecticides remain effective in MBD control and prevention.

Finally, to complicate matters, we need to face the fact that different mosquito species—and even genera—that we are currently not monitoring (or are collecting but not testing for diseases) may transmit common and emerging diseases in the State. For example, it has been shown that *Culex* mosquitoes can play a role in *Aedes*-borne disease transmission and vice versa. The same has been demonstrated between *Culex* and *Psorophora* or *Anopheles* species. Surveillance programs tend to primarily focus on, e.g., *Aedes* species when investigating diseases commonly associated with that genus (e.g., Zika, dengue). WNV is also mostly screened for in several *Culex* species, as they are considered the primary vectors. This focused approach may lead to the potential oversight of other competent vector species, which can lead to vector control decisions that do not necessarily impact disease transmission dynamics in local communities. Mosquito surveillance programs could screen for diseases in other potential vector species, even if it is in a so-called “snap-shot entomological surveillance” approach (i.e., only at certain time intervals and in certain places) [162].

Establishing a central mosquito biobank with a subset of specimens that are collected throughout the State is warranted. This will serve as a repository that will greatly enhance surveillance programs by (i) providing a central location for long-term mosquito preservation whereby specimens are available for training public health officials in species identification and (ii) serving as a valuable resource for (future) genetic studies (e.g., sequencing) to look for, e.g., insecticide resistance markers and novel viruses, or conduct species-specific molecular identification [168].

## 5. Conclusions

In this study, we presented a comprehensive and up-to-date list of all known mosquito species identified in Arizona to date. This supersedes the most recent lists published about 50 years ago. We provided valuable insights into the mosquito fauna of Arizona and shed light on the known feeding preferences of the different species, as well as the diseases they can potentially carry and/or transmit to humans and animals. To effectively address the public health implications of MBDs in the State, a large group of stakeholders—including entomologists, epidemiologists, social scientists, wildlife ecologists, and ornithologists—must collaborate and develop a mosquito surveillance framework that is tailored to better identify the factors that govern the diseases that (i) currently circulate in the State and (ii) may emerge here in the near future. Social scientists can play an important role in MBD prevention efforts by providing insights into the link between MBDs and socioeconomic status in order to identify locations or specific populations that are more at risk for MBDs. For example, studies have shown that lower socio-economic status is linked with higher disease incidence [169,170], although the opposite is observed as well [155]. The agricultural sector should also be involved if they use (or have used) insecticidal classes that are also used in public health [171,172]. Furthermore, it is paramount to collaborate with irrigation and drainage districts [173], as the systems currently in place in our desert environment may unintentionally create productive mosquito breeding areas (e.g., after flood irrigation, and inside storm drains). Additionally, it is essential to engage the community (through, e.g., community-based integrated vector management IVM programs [174]) to help with eliminating and preventing stagnant water sources serving as breeding grounds for mosquitoes. Furthermore, through collaborations with institutions like the USDA, local public health professionals can be effectively trained to monitor and detect not only autochthonous MBDs but also MBD occurrence through importations and in local zoonotic reservoirs (rodents, reptiles, amphibians, birds, etc.). This interdisciplinary approach of different stakeholders working together will ensure that proactive measures are put in place to safeguard the health of our local communities.

## Figures and Tables

**Table 5:** List of *Orthopodomyia* species.

Collection Year	Collection Method	Collection Area (by County)	Feeding Preference	Diseases They Can/May Transmit
***Orthopodomyia kummi* Edwards 1939**				
1958–1964 (UAIC)	Unknown	Santa Cruz	Unknown	Unknown
On or before 1973 [23]	Unknown	Santa Cruz		
***Orthopodomyia signifera* (Coquillett 1896)**				
On or before 1956 [61]	Unknown	Santa Cruz	Unknown	Eastern equine encephalitis and Western equine encephalitis [142]
1964–1968 (UAIC)	Unknown	Pima Santa Cruz		
On or before 1973 [23]	Unknown	Pima Santa Cruz		

**Table 6:** List of *Psorophora* species.

Collection Year	Collection Method	Collection Area (by County)	Feeding Preference	Diseases They Can/May Transmit
***Psorophora confinnis* (Lynch Arribálzaga 1891)** **^♦^** ^,^ *				
On or before 1956 [61]	Unknown	Cochise Graham Greenlee Maricopa Pima Pinal Yavapai Yuma	Large mammals [143]	Venezuelan equine encephalitis virus [143] ^a, †,†††^
On or before 1973 [23]	Unknown	Cochise Graham Greenlee Maricopa Pima Pinal Santa Cruz Yavapai Yuma		
2019 (AZDHS)	A variety of CO_2_ traps and/or BG-Sentinel and/or Oviposition traps	Yuma		
*Psorophora columbiae* (Dyar & Knab 1906) *				
1936–2022 (UAIC)	Unknown	Cochise La Paz Maricopa Pima Pinal Yuma	Humans [146] Large mammals [147]	West Nile virus [116,144] ^b, †††^ Dog heartworm [145] ^b, †††^
2010, 2012–2017, 2019–2021(AZDHS)	A variety of CO_2_ traps and/or BG-Sentinel and/or Oviposition traps	Pima Greenlee Maricopa Mohave Yuma Cochise Pinal Santa Cruz La Paz Graham Yavapai Coconino		
2016–2022 (NEON)	CDC CO_2_ light traps baited with dry ice	Pima		
***Psorophora ciliata* (Fabricius 1794)** **^♦^**				
2016–2017 (AZDHS)	A variety of CO_2_ traps and/or BG-Sentinel and/or Oviposition traps	Mohave Pima	Mammals [148]	Unknown
***Psorophora discolor* (Coquillett 1903)**				
On or before 1956 [61]	Unknown	Cochise Santa Cruz	Humans and animals [23]	Unknown
On or before 1973 [23]	Unknown	Cochise Santa Cruz		
2014–2017, 2019 (AZDHS)	A variety of CO_2_ traps and/or BG-Sentinel and/or Oviposition traps	Pima		
2014–2021 (UAIC)	Unknown	Pima		
2018 (NEON)	CDC CO_2_ light traps baited with dry ice	Pima		
***Psorophora howardii* Coquillett 1901**				
On or before 1956 [61]	Unknown	Pinal	Humans [23]	Unknown
On or before 1973 [23]	Unknown	Pinal		
1997–2021 (UAIC)	Unknown	Pima		
2012, 2014–2017, 2019 (AZDHS)	A variety of CO_2_ traps and/or BG-Sentinel and/or Oviposition traps	Pima Cochise Santa Cruz		
2022 (NEON)	CDC CO_2_ light traps baited with dry ice	Pima		
***Psorophora signipennis* (Coquillett 1904)**				
1936–2022 (UAIC)	Unknown	Cochise Graham Greenlee Navajo Pima Pinal Yavapai	Unknown	West Nile virus [144] ^b, †††^
On or before 1956 [61]	Unknown	Collected in every county except Apache, Gila, Navajo and Yuma		
On or before 1973 [23]	Unknown	Cochise Coconino Graham Greenlee Maricopa Mohave Pima Pinal Santa Cruz Yavapai Yuma		
2012–2017, 2019 (AZDHS)	A variety of CO_2_ traps and/or BG-Sentinel and/or Oviposition traps	Pima Yuma Maricopa Yavapai Greenlee Gila Graham Navajo Cochise Mohave		
2016–2018, 2021–2022 (NEON)	CDC CO_2_ light traps baited with dry ice	Pima		

**^♦^** One or more scientists in the State have expressed doubts about the accuracy of this collection record. * The southwestern *Ps. columbiae* was elevated to full species status and a Dyar name, *Psorophora toltecum (Dyar and Knab 1906)* was resurrected for it. Also, South American populations are considered *Psorophora confinnis* [149]. ^a^ Vector competence study under laboratory conditions. ^b^ Virus isolated from field-collected specimens. ^†^ Detection of virus in the saliva of mosquito post-infection. ^†††^ Disseminated infection (i.e., detection of virus in body, legs, and/or wings of mosquito).

**Table 7:** List of ***Toxorhynchites and Uranotaenia*** species.

Collection Year	Collection Method	Collection Area (by County)	Feeding Preference	Diseases They Can/May Transmit
***Toxorhynchites moctezuma* (Dyar & Knab 1906)**				
1955–2011 (UAIC)	Unknown	Cochise Pima Pinal Santa Cruz	Nectar and sugar [150]	Unknown
2017 (AZDHS)	A variety of CO_2_ traps and/or BG-Sentinel and/or Oviposition traps	Coconino		
***Uranotaenia anhydor* Dyar 1907**				
On or before 1956 [61]	Unknown	Cochise	Unknown	Unknown
***Uranotaenia sapphirina* (Osten Sacken 1868)** **^♦^**				
2015 (AZDHS)	A variety of CO_2_ traps and/or BG-Sentinel and/or Oviposition traps	Pima	Unknown	Unknown

**^♦^** One or more scientists in the State have expressed doubts about the accuracy of this collection record.

## Data Availability

The raw data from AZDHS and UAIC can be made available by the authors on request. The original data from NEON presented in this study is openly available in the NEON Biorepository Data Portal.
